# Annotations

**Published:** 1903-06-06

**Authors:** 


### Annotations.

### A Dry Bread Diet?

Mr. T. Thatcher sends to *Public Opinion* a letter which he entitles "Dr. Hardcrust," in praise of a diet of dry bread with a well baked crust. He himself recently walked forty miles in one day with no more sustenance than twopennyworth of "hard, dry brown bread crusts," and that at the age of sixty-four, and was none the worse for it, but the reverse; while a holiday in which he ate the normal quantity of "good" food resulted in nightmare and subsequent depression. Naturally a man praises the food which agrees with him, but whether or not a crust of bread be as wholesome for others as it is for Mr. Thatcher, there is no doubt that this plea for food that demands some effort in the eating is not untimely in an age when everybody seem to want to have their digestion done for them. For some people, these pre-digested foods cause an absolute hunger dyspepsia. There is nothing in them to "stay" the stomach, and the sufferer would probably be better, instead of trying to find something still more digestible, to put himself for a time on a diet of brown bread and haricot beans. Not that either of these is indigestible, though some might think the latter was. It is, indeed, difficult of digestion, but when digested the food-value is high. The really indigestible things are those which have little food value, and make the stomach work for no profit; but if it be worth the effort, a food that is difficult of digestion has a certain merit of its own, in that it exercises the organs of digestion. The gourmet dyspeptic who resented the simple strengthening fare we speak of might console himself with the thought that when his digestion was thus strengthened, he would be able to enjoy the pleasures of the table more than ever before, but if in the process of treatment he grew to enjoy the simplest foods, he would be none the loser.

### The Royal Society and the New Lymph.

After the wonder-waking surprises to which the Royal Society has treated its Conversazione guests on several occasions during late years, the exhibits which it had contrived to collect for the recent spring festa seemed somewhat tame. Certainly, to all medical men by far the most interesting exhibit was that of Dr. Alan B. Green, and the general public would have been not less interested had it appreciated the fact that it was present at the first public appearance of a vaccine-lymph with which before long it is likely to have an intimate personal experience. Vaccine lymph, of course, as drawn from the calf is anything but pure, and is unfit for use until it has been sterilised as regards all organisms other than the essential entity which constitutes vaccine. At present glycerine is used for this purpose. It is effective, but it takes some four and sometimes more weeks to complete the process, and there is always a risk in epidemic times of vaccine being sent out before it is absolutely sterile. This is certainly rarely the case, but many medical men must have seen arms which have suggested to them a doubt as to whether the vaccine they used was as perfect as it might be. The new process, however, is extremely rapid, and in little over six hours lymph freshly drawn from the calf can be issued absolutely sterile of all micro-organisms capable of being cultivated, but retaining its full vaccine potency. The *modus operandi* is as follows: The lymph is first rubbed up lightly with freshly-sterilised water so as to form a thin emulsion. Then sterilised air is submitted to the vapour of chloroform and thereafter allowed to pass upwards through the emulsion. The water of the latter thus becomes saturated with chloroform to the extent of about 1 in 200. After an exposure of about six hours the chloroform is allowed to evaporate, and on testing by agar plate culture the emulsion is found to be sterile. Plate cultures of vaccine prepared by both methods were exhibited side by side, and the difference in the results was very striking.

### Clay Modelling and Osteology.

The difficulty of giving an adequate representation by words of objects with three dimensions is not peculiar to the child who portrayed the horse as a square animal with a leg at each corner. It is a failing which is common to the human race, and it is one of the problems which those who interest themselves most in educational matters have from time to time attempted to solve. To the normal human being one good illustration can convey more meaning than a whole chapter of verbal description by itself, and the value of the latter lies for the most part in calling attention to the salient features of an object which is before the reader or of which he has a good reproduction to refer to. But even pictures fall short of the ideal, for they cannot do justice to the third dimension. To nobody are these facts of more significance than to the student of anatomy, who has to some extent to develop his knowledge from text-books. The mental effort required to appreciate the accounts given in the books in the absence of the object described produces a nervous exhaustion which is out of all proportion to the information obtained. Again, when it comes to an examination, the art of writing an anatomy paper requires something apart from a knowledge of anatomy, and so it comes about that a student who is well up in objective anatomy, if one may call it so, may be quite out-classed in an examination paper by a mere book-worm who knows his text-book well, although in the practical application of his knowledge the latter may be far the inferior. The Americans have been the first to make a serious attempt to overcome these difficulties. In some of their universities they have recently adopted a method of clay-modelling for students of osteology. The learner makes models in clay of the different bones, first with the object before him and eventually from memory. This plan seems quite an ideal one for learning bones. No eye-strain or mental exhaustion is called for, manual dexterity is acquired, and finally the examination of the student's knowledge by setting him to make reproductions of the various bones is easy and satisfactory. It is greatly to be desired that the English anatomy schools will make a trial of the above method of teaching osteology, for thereby a great advance will be made and an example set which might with the greatest advantage be extended to other branches of education.

